# The Prevalence and Risk Factors for Gallstone Disease in Taiwanese Vegetarians

**DOI:** 10.1371/journal.pone.0115145

**Published:** 2014-12-18

**Authors:** Yen-Chun Chen, Chia Chiou, Ming-Nan Lin, Chin-Lon Lin

**Affiliations:** 1 Department of Internal Medicine, Dalin Tzu Chi Hospital, Buddhist Tzu Chi Medical Foundation, Chiayi County, Taiwan; 2 Department of Family Medicine, Dalin Tzu Chi Hospital, Buddhist Tzu Chi Medical Foundation, Chiayi County, Taiwan; 3 Department of Family Medicine, College of Medicine, Tzu Chi University, Hualien, Taiwan; 4 Medical Mission, Tzu Chi Foundation, Hualien, Taiwan; 5 Department of Internal Medicine, Buddhist Hualien Tzu Chi Hospital, Hualien, Taiwan; 6 Department of Internal Medicine, College of Medicine, Tzu Chi University, Hualien, Taiwan; National Institute for Viral Disease Control and Prevention, CDC, China, China

## Abstract

**Introduction:**

Gallstone disease (GSD) and its complications are major public health issues globally. Although many community-based studies had addressed the risk factors for GSD, little is known about GSD prevalence and risk factors among Taiwanese vegetarians.

**Methods:**

This study included 1721 vegetarians who completed a questionnaire detailing their demographics, medical history, and life-styles. GSD was ascertained by ultrasonography or surgical history of cholecystectomy for GSD. The predictive probability of GSD for male and female vegetarians was estimated from the fitted model.

**Results:**

The prevalence of GSD was 8.2% for both male and female vegetarians. The risk of GSD is similar in men and women across all age groups, and increases steadily with increasing age. For male vegetarians, age (OR: 1.04; 95% CI: 1.00–1.08) and serum total bilirubin level (OR: 2.35; 95% CI: 1.31–4.22) predict risk for GSD. For female vegetarians, age (OR: 1.03; 95% CI: 1.01–1.05), BMI (OR: 1.07; 95% CI: 1.01–1.13), and alcohol consumption (OR: 7.85; 95% CI: 1.83–33.73) are associated with GSD. GSD is not associated with type of vegetarian diet, duration of vegetarianism, low education level, physical inactivity, diabetes, coronary artery disease, cerebral vascular accident, chronic renal failure, hepatitis C virus infection, and lipid abnormalities. GSD is also not associated with age at menarche, postmenopausal status, and multiparity in female vegetarians.

**Conclusions:**

Risk factors useful for predicting GSD in vegetarians are (1) age and total bilirubin level in men, and (2) age, BMI, and alcohol consumption in women. Many previously identified risk factors for general population does not seem to apply to Taiwanese vegetarians.

## Introduction

The burden of gallstone disease (GSD) and its complications, such as cholecystitis, pancreatitis, and cholangitis, are major public health issues globally [1]. A 2006 study reported that more than 700,000 cholecystectomies were performed in the United States at a cost of $6.5 billion dollars annually [1]. Most patients with GSD are asymptomatic, and approximately 20% become symptomatic after 10 years of follow-up [2]. Ultrasonography is recognized as the gold standard for diagnosing GSD [3].Ultrasonography-based studies showed that the cumulative incidence rate of GSD is 0.67% per year (0.66% in males, 0.81% in females) in Italians and the incidence rate is 1.39 per 100 person-years in Sweden [4], [5].

The prevalence of GSD varies among different populations. Westerners tend to have higher prevalence than Asians: 16.6% and 8.6% in non-Hispanic white women and men in the United States respectively [6]; 14.6%–18.4% and 6.7%–9.5% in Italian women and men [4], [7]; 22.4% and 11.5% in British women and men [8], but only 10.7%, 6.6%, 5%, and 3.2% in China, Singapore, Taiwan, and Japan, respectively [9]–[12].

Previous studies have identified age, female gender, family history of gallstone, pregnancy, diabetes, and obesity as risk factors for GSD [11], [13]–[15]. One study reported an odds ratio of 1.9 for the development of GSD among nonvegetarians compared with vegetarians after adjusting for age and body mass index (BMI) in women [16]. Another study showed that greater fruit and vegetable consumption decrease the risk of cholecystectomy in women [17]. A third study with small sample size showed no significant difference in the prevalence of GSD between nonvegetarians and vegetarians [11]. Thus, how diet affects GSD remains uncertain.

This cross-sectional study aims to evaluate the gender-based prevalence and risk factors for GSD in vegetarians in Taiwan.

## Methods

### Study participants

The participants were recruited through the Tzu Chi Health Study (n = 6002), and all of them received a health examination at Buddhist Dalin Tzu Chi Hospital between October 2007 and November 2010 [18]. After excluding the nonvegetarians, and those with previous cholecystectomy due to conditions other than GSD, a total of 425 male and 1296 female vegetarians were included in this current analysis. The study was approved by the institutional review board at the Buddhist Dalin Tzu Chi Hospital, and all participants provided written informed consent before enrollment.

### Clinical assessment

All participants were interviewed by one of two trained research assistants on demographics, medical history, life-styles, and dietary patterns, through a structured questionnaire. Dietary pattern was evaluated using a validated food frequency questionnaire (FFQ) [19]. We classified vegetarians into several sub-types: lacto-ovo-vegetarian (consuming dairy products and eggs, but no other animal products), lacto-vegetarian (consuming dairy products but no other animal products), ovo-vegetarian (consuming eggs but no other animal products) and vegan (plant-based foods only).

Life-style parameters are defined as follows: Moderate alcohol consumption is defined as having more than 1 drink but less than 9 drinks for women and 12 drinks for men per week; physical inactivity is defined as less than 60 minutes per week on vigorous exercise. Education level was defined as high for participants with a bachelor's degree or higher. Height and body weight were measured, and body mass index (BMI) was calculated by dividing weight (kg) by the square of height (m^2^).

The diagnosis of GSD was ascertained by ultrasonography or having a medical history of cholecystectomy for GSD. Diabetes, hypertension, coronary artery disease (CAD), cerebral vascular accident (CVA), chronic renal failure (CRF) were identified through the self-reported medical history section in the questionnaire. Venous blood was collected after at least 8 hours of fasting and examined for serum total cholesterol (TCH), triglyceride (TG), HDL-C, LDL-C, total bilirubin level (TBL) (Dimension RXL Max integrated chemistry system, Siemens, Germany). Serum markers including HBsAg, HBsAb, and Anti-HCV Ab were used to identify infection of hepatitis B viral (HBV) and hepatitis C viral (HCV) (ARCHITECT i1000SR Immunoassay Analyzer, Abbott, USA).

### Statistical analysis

Demographic characteristics were compared using χ^2^ test for categorical variables and independent *t* test for continuous variables. Categorical values less than 5 were assessed by Fisher's exact test. A binary logistic regression analysis was performed to assess the independent influence of potential risk factors – age, BMI, education level, alcohol consumption, physical inactivity, types of vegetarians, duration of vegetarianism, menarche >16 years, multiparity, postmenopausal status, diabetes, HTN, CAD, CVA, CRF, HBV infection, HCV infection, TCH, TG, LDL-C, HDL-C and TBL – on GSD. A stepwise logistic regression was applied for the development of the fitted model estimating the predictive probability of GSD in both genders. All data were processed using SPSS version 21 (IBM, Armonk, NY, USA).

## Results

This study enrolled 1721 vegetarians (24.7% men and 75.3% women) with a mean age of 54.8±9.5 for men and 54.2±9.3 for women. Compared with men, women had a lower BMI and a higher average body fat. Alcohol consumption was low in both genders. Men had a higher education level. The GSD prevalence was 8.2% for both male and female vegetarians, as shown in Table 1. The GSD prevalence in male omnivores in the Tzu Chi Health Study is 8.3% (N = 1973), and is 7.5% (N = 2160) in female omnivores (data not shown).

**Table 1:** Demographic characteristics of subjects.

Characteristics‡	Male* 425(24.7)	Female* 1296(75.3)
Age (y)	54.77±9.5	54.17±9.3
<40	18(4.2)	61(4.7)
40–49	105(24.7)	359(27.7)
50–59	179(42.1)	544(42.0)
60–69	94(22.2)	246(19.0)
≥70	29(6.8)	86(6.6)
BMI (kg/m^2^)	23.4±2.9	22.9±3.1
Education(bachelor's degree or higher)	130(30.6)	223(17.2)
Alcohol consumption	5(1.2)	8(0.6)
Physical inactivity	155(36.5)	530(40.9)
Menarche>16 y		354(27.3)
Multiparity		257(19.8)
Postmenopausal status		896(69.1)
Diabetes	24(5.6)	46(3.5)
Hypertension	77(18.1)	211(16.3)
CAD	16(3.8)	65(5.0)
CVA	5(1.2)	5(0.4)
CRF	1(0.2)	4(0.3)
HBV infection	86(20.2)	196(15.1)
HCV infection	17(4.0)	68(5.2)
TCH (mg/dL)	173.3±35.0	183.7±32.8
TG(mg/dL)	126.8±89.7	111.0±74.4
HDL-C (mg/dL)	45.1±10.7	55.1±13.8
LDL-C(mg/dL)	113.7±29.2	117.4±29.6
TBL(mg/dL)	1.0±0.5	0.7±0.3
Gallstones	35(8.2)	106(8.2)

*Data shown as number (%) or mean ± SD.

‡ BMI  =  body mass index; CAD  =  coronary artery disease; CVA  =  cerebral vascular accident; CRF  =  chronic renal failure; HBV  =  hepatitis b virus; HCV  =  hepatitis c virus; TCH  =  total cholesterol; TG  =  triglyceride; HDL-C  =  high-density lipoprotein cholesterol; LDL-C  =  low-density lipoprotein cholesterol; TBL  =  total bilirubin level.

The prevalence of GSD in different age groups are reported in Table 2. The risk of GSD is similar between men and women, and both show an increasing trend with increasing age (P for trend <0.001 in men and women). For men, age (OR: 1.04, *P* = 0.052) was weakly associated with GSD. Other demographic and life styles characteristics, such as BMI, type of vegetarian, duration of vegetarianism, education level, alcohol consumption, and physical inactivity were not related to GSD. For women, age (OR: 1.03, *P* = 0.052) and BMI (OR: 1.06, *P* = 0.064) were weakly associated with GSD. Compared with abstinence, moderate alcohol consumption emerged to be a strong risk factor (OR 7.61, *P* = 0.007) for GSD in women. Hormone-related factors and gynecological conditions such as age at menarche >16 years, multiparity, and postmenopausal status were not associated with GSD (Table 3).

**Table 2:** Prevalence of GSD§ in different age groups(by sex).

Age	Men(N/%)	Women(N/%)	*P-*value
<40	0(0)	2 (3.3)	1.000*
40–49	7 (6.7)	19 (5.3)	0.630
50–59	16 (8.9)	52 (9.6)	0.883
60–69	8 (8.5)	23 (9.3)	1.000
≥70	4 (13.8)	10 (1.6)	0.749
Overall	35 (8.2)	106 (8.2)	0.971
Age-adjusted prevalence‡	(3.02)	(4.49)	0.203

§ GSD = gallstone disease.

*Fisher's exact test.

‡ Age-standardised to World Standard Population (WHO 2000–2025).

**Table 3:** The influence of demographics, life styles, and hormone-related factors on GSD§.

	Men		Women		Overall	
Characteristics‡	OR (95% CI)	*P* value	OR (95% CI)	*P* value	OR (95% CI)	*P* value
Age	1.04(1.00–1.08)	0.052	1.03(1.00–1.07)	0.052	1.03(1.01–1.05)	0.007
BMI	1.02(0.90–1.15)	0.764	1.06(1.00–1.13)	0.064	1.05(0.99–1.11)	0.071
Types of vegetarians						
Ovo-Lacto vs Vegan	0.69(0.14–3.34)	0.643	0.87(0.33–2.33)	0.787	0.78(0.34–1.78)	0.556
Lacto vs Vegan	1.56(0.25–9.88)	0.639	0.77(0.24–2.53)	0.669	0.91(0.34–2.44)	0.843
Ovo vs Vegan	0.39(0.05–3.17)	0.375	0.93(0.28–3.09)	0.904	0.74(0.26–2.08)	0.568
Duration of vegetarianism (y)						
5–15 vs<5	0.82(0.36–1.86)	0.638	1.26(0.77–2.09)	0.361	1.12(0.73–1.70)	0.612
>15 vs<5	0.53(0.20–1.43)	0.210	1.07(0.59–1.95)	0.831	0.88(0.53–1.46)	0.618
Low education level	1.36(0.59–3.15)	0.478	1.42(0.74–2.71)	0.289	1.38(0.83–2.27)	0.212
Moderate alcohol consumption	0.00(0.00–0.00)	0.999	7.62(1.72–33.71)	0.007	3.49(0.93–13.11)	0.064
Physical inactivity	1.07(0.50–2.27)	0.867	1.01(0.66–1.55)	0.959	1.02(0.71–1.47)	0.924
Menarche>16 y			0.81(0.50–1.31)	0.396		
Multiparity			1.35(0.82–2.24)	0.243		
Postmenopausal status			0.73(0.40–1.34)	0.306		

§ GSD = gallstone disease.

‡ BMI  =  body mass index; Physical inactivity: less than 60 minutes per week on vigorous exercise; low education level: education level below the bachelor's degree; multiparous: procreation >3.

For men, serum total bilirubin level was a stronger risk factor (OR 2.34, *P* = 0.004) after adjustment for age and BMI. Hypertension was associated with GSD (OR = 0.30, *P* = 0.055), though this association did not reach statistical significance. Diabetes, CAD, CVA, CRF, HBV infection, HCV infection, and all types of lipid profiles were not associated with GSD as shown in Table 4. Diabetes (OR 2.50, p<0.001) and total bilirubin level (OR 1.52, p = 0.010) were associated with GSD in male omnivores in the Tzu Chi Health Study (data not show).

**Table 4:** Associations among systemic diseases, lipid profiles, bilirubin level, and GSD§ adjusted by age and BMI*.

	Men		Women		Overall	
Characteristics‡	OR (95% CI)	*P* value	OR (95% CI)	*P* value	OR (95% CI)	*P* value
Diabetes	1.46(0.41–5.24)	0.561	1.03(0.39–2.71)	0.953	1.16(0.54–2.50)	0.712
Hypertension	0.30(0.09–1.02)	0.055	1.20(0.72–2.02)	0.482	0.90(0.56–1.43)	0.655
CAD	1.08(0.22–5.35)	0.923	0.67(0.26–1.75)	0.416	0.77(0.34–1.73)	0.523
CVA	0.00(0.00–0.00)	0.999	2.59(0.28–23.70)	0.399	0.99(0.12–7.92)	0.990
CRF	0.00(0.00–0.00)	1.000	2.96(0.30–29.51)	0.355	2.51(0.27–23.27)	0.419
HBV infection	1.66(0.73–3.79)	0.999	1.58(0.96–2.61)	0.074	1.61(1.05–2.46)	0.029
HCV infection	0.00(0.00–0.00)	0.999	0.95(0.40–2.26)	0.900	0.74(0.32–1.74)	0.491
TCH	0.99(0.98–1.01)	0.292	1.00(0.99–1.01)	0.753	1.00(0.99–1.00)	0.433
TG	1.00(0.99–1.00)	0.401	1.00(0.99–1.00)	0.656	1.00(0.99–1.00)	0.383
HDL-C	1.00(0.97–1.04)	0.969	1.00(0.98–1.01)	0.759	1.00(0.98–1.01)	0.851
LDL-C	0.99(0.98–1.01)	0.568	1.00(0.99–1.01)	0.918	1.00(0.99–1.01)	0.848
TBL	2.34(1.30–4.21)	0.004	0.95(0.50–1.83)	0.886	1.41(0.94–2.11)	0.101

§ GSD = gallstone disease.

*BMI = body mass index.

‡ CAD  =  coronary artery disease; CVA  =  cerebral vascular accident; CRF  =  chronic renal failure; HBV  =  hepatitis b virus;

HCV  =  hepatitis c virus; TCH  =  total cholesterol; TG  =  triglyceride; HDL-C  =  high-density lipoprotein cholesterol; LDL-C  =

low-density lipoprotein cholesterol; TBL  =  total bilirubin level.

The probabilities of GSD in men and women were estimated using stepwise logistic regression analyses. Potential risk factors for GSD discussed in the current study were used for estimating the fitted model. The fitted model for the probability of GSD in men was:

*P* = *e* ^–5.332+[0.037•Age]+[0.854•Serum total bilirubin level]^/1+*^e^* ^–5.332+[0.037•Age]+[0.854•Serum total bilirubin level]^, where *P* was the probability of detecting GSD in male vegetarians (Table 5).

**Table 5:** Stepwise logistic regression with respect to GSD§ in vegetarians.

Factors‡	OR(95% CI)	P value
Men		
Age (y)	1.04(1.00–1.08)	0.053
Total bilirubin level	2.35(1.31–4.22)	0.004
Women		
Age	1.03(1.01–1.05)	0.011
BMI*	1.07(1.01–1.13)	0.044
Alcohol consumption	7.85(1.83–33.73)	0.006

§ GSD = gallstone disease.

*BMI = body mass index.

‡ Dependent variable: GSD; independent variables: age, BMI, total bilirubin level, and alcohol consumption.

The fitted model in women was:

*P* = *e* ^–5.432+0.028•Age+0.063•BMI+[2.061, if consuming>1 drink/week]^/1+*e* ^–5.432+0.028•Age+0.063•BMI+[2.061, if consuming>1 drink/week]^, where *P* was the probability of detecting GSD in female vegetarians.

**Figure 1:**
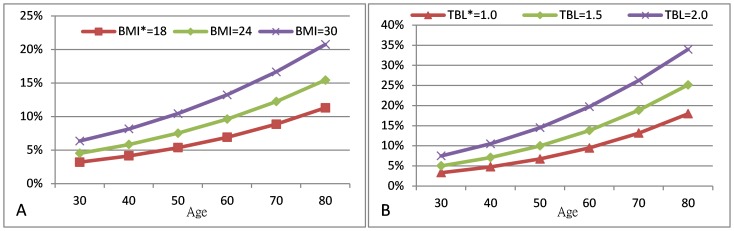
The predictive probability of GSD^§^ in male vegetarians and female vegetarians without alcohol consumption. A: female vegetarians without alcohol consumption; B: male vegetarians. ^§^ GSD = gallstone disease ^*^ BMI = body mass index; TBL = serum total bilirubin level.

The predicted probabilities of GSD estimated from the fitted model are shown in Fig. 1. For male vegetarians, the probability of GSD increased with age and serum bilirubin levels. For female vegetarians, age, BMI and alcohol consumption were associated with increasing probability for GSD.

## Discussion

For Taiwanese vegetarians, types of vegetarians and the duration of vegetarianism are not related to the risk of GSD. The risk factors of GSD vary with gender: age and elevated total bilirubin level for men, and age, BMI, and alcohol consumption for women. Age, BMI, diabetes, lower HDL-C, and glucose intolerance are risk factors for GSD in Taiwanese population [11], [20], [21]. This study demonstrates that the effect of risk factors on GSD may be influenced by dietary patterns.

The prevalence rate of GSD in this prospective cross-sectional study is 8.2% which is higher than one community-based study (5.0%) of middle aged adults in Taiwan but lower than another hospital-based study (10.7%) with elderly subjects [11], [20]. The difference may be explained by the age distribution and the selection of subjects studied. Among adults >60 years old in this study, the GSD prevalence was 13.8%, which is similar to previously reported value of 10%–16.6% in Taiwan [11], [20], [21]. Vegetarian diets are associated with decreased risk for GSD [16], [22], [23]. However, the GSD prevalences in omnivores and vegetarians in the Tzu Chi Health Study are similar. The omnivores in the Tzu Chi Health Study had been encouraged to consume more fiber and less meat, therefore the effect of diet on GSD may be weakened.

In the present study, increasing age was weakly associated with GSD in both genders. The association between age and GSD has been reported in previous Taiwanese studies [20], [21]. This study demonstrates that BMI is not related to the rate of GSD in male vegetarians but is weakly associated with GSD in females. BMI has been reported to be associated with GSD in one hospital-based study but the correlation has not been observed in another study [20], [21]. The age and sex distribution of subjects may contribute to the difference.

The prevalence of GSD in male and female vegetarians is similar in this study. Gender is reported to be risk factor for GSD [4], [6]. Some Asian studies have showed a higher frequency of GSD in women than in men, but the difference is not as large as in Western populations [9], [10]. Nevertheless, the difference in the prevalence of GSD between sexes is not significant in Taiwan [11], [21].

Types of vegetarians and duration of vegetarianism are not associated with GSD in this study. In the Adventist Health Study-2 (AHS-2), vegans have been previously reported to have lower BMI (23.6 kg/m^2^) than lacto-ovo vegetarians (25.7 kg/m^2^), and pesco-vegetarians (26.3 kg/m^2^), and lower BMI may prevent GSD. [24], [25] However, vegans in AHS-2 also had lower daily intake of calcium (610 mg) than lacto-ovo vegetarians (1087 mg), fish-eaters (1081 mg), and meat-eaters (1057 mg) and calcium intake is reported to be inversely associated with GSD incidence [26], [27]. The association between duration of vegetarianism and GSD has not been discussed previously and more studies are needed to verify the correlation between types of vegetarians, duration of vegetarianism and GSD.

Multiparous women did not show a significantly higher rate of GSD in this study. Pregnancy is an important pathogenic factor for GSD. Biliary sludge and gallstones in some women were found to disappear after delivery [28]. Another study reported that multiparous females had a higher prevalence of GSD than nulliparous ones [29]. This difference may be explained by the ethnicity factors. Other factors influencing female hormones, such as age at menarche and postmenopausal status, were not associated with the risk of GSD in the present study, and this is consistent with findings in previous studies [30], [31].

This study suggests that moderate alcohol consumption is strongly associated with GSD in female but not males vegetarians. Moderate alcohol consumption has been demonstrated to be negatively associated with GSD in previous studies [21], [32]–[35]. All drinkers in this study were moderate drinkers but the number of drinkers was small and may lead to a bias. Two reports suggested that physical activities may be beneficial to prevent symptomatic GSD or cholecystectomy [36], [37]. This study focused mainly on asymptomatic GSD and suggests that physical activities are not associated with the risk of GSD in vegetarians.

This study revealed that DM, CAD, CVA, and CRF are not associated with GSD in male and female vegetarians. In some community-based studies, diabetes mellitus (DM) has been identified as a risk factor for GSD in both men and women or in women only [20], [21], [38]. The difference may be explained by the protective effect of vegetarian diets. This study also revealed no association between serum lipid profiles and GSD, which is consistent with results from previous studies [20], [39].

In this study, elevated total bilirubin levels were significantly associated with GSD in male vegetarians but not in female vegetarians. A community-based study in Denmark has showed that elevated bilirubin level is a causal risk factor for symptomatic GSD [40]. This study found that both hepatitis B and hepatitis C viral infection were not related to GSD in male and female Taiwanese vegetarians. The correlations between HBV infection, HCV infection and GSD remain controversial. In previous community-based studies, chronic HCV infection is related to GSD among men but not women in the United States and both HBV infection and HCV infection are correlated with GSD in either sex in Taiwan. [41], [42]. However, one hospital-based study and another community-based study from Taiwan showed no correlation between HBV infection, HCV infection and GSD [11], [21].The discrepancy may be explained by the gender difference, ethnicity factors and the selection of subjects studied.

### Limitation

There are some limitations in the present study. First, the subjects in this study were mostly Tzu Chi commissioners - a devoted group of volunteers of the Buddhist Tzu Chi Foundation. The subjects are highly homogeneous and may not represent the general population in Taiwan, but due to the high proportion of vegetarians, it provides an excellent opportunity to examine risk factors for GSD among vegetarians. Secondly, some participants with dyslipidemia may have used lipid lowering agents and this could potentially contribute to the null association between GSD and dyslipidemia.

Our findings suggest that the risk factors for GSD in vegetarians vary with gender. Age is a major and universal risk factor for GSD. Elevated total bilirubin level and BMI also emerged to be risk factors in male and female vegetarians, respectively. In vegetarians, the types of vegetarians and duration of vegetarianism play no role in GSD.
